# Supplementary material for: Proteomics Analysis of Peripheral Blood Mononuclear Cells from Patients in Early Dengue Infection Reveals Potential Markers of Subsequent Fluid Leakage
Source: Viruses. 2025 May 31;17(6):805. doi: 10.3390/v17060805 (PMC12197526; doi:10.3390/v17060805)
Supplement: Supplementary file 1 [file viruses-17-00805-s001.zip › Supplementary File S1.pdf]

Supplementary File S1: Materials and Methods

Measurement of viral load in serum

RNA extraction of serum samples was performed using the QiaAmp viral RNA mini kit (Qiagen) using 140 µL of serum according to the manufacturer's instructions. The extracted RNA was reverse-transcribed to cDNA using the high-capacity cDNA reverse transcription kit (ThermoFisher Scientific). Following reverse transcription, 2 µL of cDNA was used in a 20 µL qRT-PCR reaction which comprised 10 µL Taqman multiplex mastermix (ThermoFisher Scientific), 900 nM of each primer and 250 nM of each probe to amplify and measure DENV copies. The standard curve was generated by a mixture of gBlock DNA fragments similar to the sequences amplified in the PCR reaction, and the viral load in the sample was calculated using the standard curve.

The DENV serotype was identified and the viral load was measured by a multiplex qRT-PCR using four different Taqman primers and dual labelled probes for the four serotypes. All probes were labelled with the QSY quencher. DENV targets and primers used for quantifying viral load by qRT-PCR are given in Table 2.

Supplementary Table S3: Target genes and the primer sequences used for qRT-PCR of DENV RNA.

Target	Forward primer (5'-3')	Reverse primer (5'-3')
DENV 1 NS5	CAAAAGGAAGTCGYGCAATA	CTGAGTGAATTCTCTCTRCTRAAC
DENV 2 E	CAGGYTATGGCACYGTCACGAT	CCATYTGCAGCARCACCATCTC
DENV 3 prM	GGACTRGACACACGCACYC	CATGTCTCTACCTTCTCGACTTGYCT
DENV 4 prM	TTGTCCTAATGATGCTRGTCG	TCCACCYGAGACTCCTTCCA

PBMC isolation

The PBMCs were isolated by a gradient centrifugation method. Whole blood (5 ml) was collected in a heparinised 15 ml falcon tube and transported to the laboratory at room temperature for PBMC isolation within 4 hours. Blood was diluted 1:1 in sterile phosphate-buffered saline (PBS) and overlayed slowly onto 5 ml of Lymphoprep (STEMCELL Technologies). The sample was centrifuged at 2000 rpm (900g) at room temperature for 20 min, with the brake off. The PBMC layer was aspirated using a sterile Pasteur pipette and transferred to a new tube. Ice-cold PBS was added to the cells and they were centrifuged at 1500 rpm (500g), for 10 min at 4 °C, with the brake on low. The supernatant was discarded and the cell pellet was resuspended in 1x red blood cell lysis buffer (BioLegend) in deionized water and incubated at 4°C for 5 min to remove any red cells. The lysis buffer was removed by adding 14 ml PBS and centrifuged at 960 rpm (200g) for 10 min at 4°C, with the brake on high. Cells were washed once in PBS and lysed to generate cell lysates for mass spectrometry.

Tandem mass tagging

PBMC cell lysates were produced by resuspending the cell pellets in RIPA buffer, supplemented with protease inhibitor (Roche) and phosphatase inhibitor (Sigma), and incubating them at 4°C for 20 min. The lysates were centrifuged at 12,000g for 10 min and the supernatant was collected for experiments. The lysates were subjected to quantification of protein levels by the Pierce BCA protein assay kit (ThermoFisher Scientific). The four DF and four DHF samples with the highest protein concentrations present were selected for tandem mass tagging (TMT). The protein amount was adjusted to 7.5 μg for each sample and was run for 5 min (approximately 5 mm) into a 4-12% Bis-Tris 10-well SDS-PAGE gel using the MOPS buffer system (ThermoFisher Scientific). The gel was stained with InstantBlue protein stain (Exedeon)

and the bands were excised from the gel with a scalpel and transferred to fresh tubes. In-gel trypsin digestion was carried out as previously described (33) using sequencing-grade porcine trypsin (Promega). Further normalisation was performed by determining the peptide concentrations of the samples using the Pierce quantitative colorimetric peptide assay (ThermoFisher Scientific). Labelling was carried out using the TMT10plex isobaric labelling kit (ThermoFisher Scientific) according to the manufacturer's recommended protocol. The labels for the 10-plex kit were used with the following tags in parentheses: two healthy donors (126, 127N), four DF patients (127C, 128N, 128C, 129N) and four DHF (129C, 130N, 130C, 131) patients, where the number refers to the mass of the reporter in Daltons and if two tags have the same mass, N indicates if the nitrogen in the mass reporter is a heavy stable isotope, and C indicates if only carbons are heavy labelled in the mass reporter. Each TMT 10-plex tag (19 μl) was mixed with 50 μl of each normalised peptide sample and then 60 μl of each of these 10 samples was pooled. This pooled sample (600 μl) was dried in a Concentrator Plus centrifugal evaporator (Eppendorf) and then resuspended in 300 μL of 0.1%v/v TFA. As a check for labelling, 5 μl of this unfractionated sample was run through liquid chromatography–mass spectrometry (LC-MS) and the data was searched on the Mascot software as described below. After confirming reliable labelling efficiency, the remaining 295 μL of pooled sample was fractionated into 11 fractions using the Pierce high-pH reversed-phase peptide fractionation kit (ThermoFisher Scientific) according to the manufacturer's recommended protocol. These 11 fractions were run through LC-MS (25 μl for fractions 1-3 and 15 μl for all other fractions) and the data was searched on the PEAKS Studio X software as described below.

Liquid chromatography

The 11 TMT labelled peptide fractions were separated on a Dionex Ultimate 3000 nano UHPLC system (ThermoFisher Scientific). A nano analytical C18 reversed-phase column (PepMap) was used (column dimensions: 75 µm diameter, 50 cm length, 2 µm particle size) (ThermoFisher Scientific) with a flow rate of 250 nL/min at 45°C. The mobile phase used was solvent A (0.1% v/v formic acid in LC-MS-grade water) and solvent B (0.1% v/v formic acid in 80% v/v acetonitrile). The gradient was used to run the peptides for the TMT labelling efficiency check as previously described (34). A longer 3-hour gradient was used to separate peptides from the 11 fractions as follows: 2% B (0-6.6 min), 2-35% B (6.6-150 min), 35-60% B (150-185 min), 60-95% B (185-186 min), 95% B (186-194 min), 95-2% B (194-195 min) and 2% B (195-210.1 min) for column equilibration.

Mass spectrometry

Peptides from the nano LC were analysed on a benchtop Q Exactive hybrid quadrupole–Orbitrap mass spectrometer using the Nanospray Flex ion source (ThermoFisher Scientific). Prior to data acquisition, the mass spectrometer was calibrated for mass accuracy according to the manufacturer's recommendations using a positive ion calibration solution injected at 5 µl/min into a heated electrospray ionisation (HESI) probe (ThermoFisher Scientific). The conditions for data-dependent acquisition (DDA) were as follows: chromatographic peak width was set at 20 s and the full MS conditions involved a resolution of 70,000, AGC target of 3e6, maximum IT (injection time) of 60 ms, and scan range of 375 to 1500 m/z. The dd-MS2 conditions were a resolution of 35,000, AGC target of 1e5, maximum IT of 60 ms, loop count of 10 (i.e., top 10), isolation window of 2.0 m/z, fixed first mass of 120.0 m/z and normalised

collision energy (NCE) of 35 in a high-energy collision dissociation (HCD) cell. The data-dependent (dd) settings included a minimum intensity threshold of 3.3e4 ions (charge exclusion: unassigned, 1, 8, >8; peptide match: preferred; dynamic exclusion: 30 s).

Protein identification

The acquired *.raw file for the 5 µl labelling check was converted to a Mascot generic file (*.mgf) using MSConvertGUI 64-bit (ProteoWizard). A peak-picking filter was set with MS levels 1-2. The Mascot server software (Matrix Science, London) was used to search the *.mgf file against the SwissProt database. The search parameters in Mascot were set as follows: MS/MS Ion Search with trypsin as the protease. Carbamidomethyl (C) and Oxidation (M) were set as variable modifications. Taxonomy was set to *Homo sapiens* (human). Peptide mass tolerance and fragment mass tolerance were both set as ± 10 ppm and the peptide charge was set as +2, +3 and +4. Data format and instrument were set as Mascot generic and Q Exactive, respectively. A decoy database was searched with the false discovery rate (FDR) adjusted to 1%. Searches were carried out with both no fixed modification and TMT as a fixed modification to calculate the labelling efficiency. The percentage of proteins labelled with TMT was calculated as follows: (Number of proteins identified with TMT 10plex ÷ Total number of proteins identified) x 100.

Once a high percentage of labelling was determined, the high-pH reversed-phase fractionation and LC-MS runs of each fraction were carried out. The 11 *.raw files for each fraction were searched on the PEAKS Studio (version X) software against the Uniprot_SwissProt database. FDR was set at <1% and precursor mass tolerance to 10 ppm. Trypsin was set as the protease, Oribtrap as the instrument, CID\HCD as the fragmentation

and MS2 as the reporter ion type. TMT was set as a fixed modification. Both Carbamidomethyl (C) and Oxidation (M) were set as variable modifications. Taxonomy was set to both human and dengue virus.

Proteomic data analysis

All human proteins identified in the samples were selected for data analysis. The average value of the MS2 tag peak intensity was calculated for proteins with multiple peptides. The mean MS2 tag peak intensity ratio for each peptide was calculated for the healthy, DF and DHF samples. The H1 healthy sample was used as the reference sample to calculate this ratio as the relative MS2 tag peak intensity for each sample. The fold change for each protein was calculated for dengue-infected samples (DF and DHF both) compared to healthy controls (H1 and H2) and DF (DF 1-4) compared to DHF (DHF 1-4) using average values for each group. A t-test was performed among samples to identify a statistically significant difference in protein expression between healthy controls and dengue-infected samples. A similar analysis was performed for DF and DHF. Proteins with a fold difference exceeding 1.5 and $p<0.05$ were selected to identify biological pathways associated with the proteins using the STRING database. Figures were generated using GraphPad Prism version 9.0 and R studio with R v4.1.2. Protein to protein interaction (PPI) networks were created using Cytoscape v3.9.1. Gene Ontology enrichment analysis was conducted by calculating probabilities and fold enrichment values from observed differently expressed proteins. The Gene Ontology (GO) database (35) was used to enrich biological processes, molecular function and cellular components, while the Kyoto Encyclopedia of Genes and Genomes (KEGG) and REACTOME databases were used to enrich pathways (36,37). Fold enrichment and false discovery rate (FDR) were calculated for each pathway. Fold enrichment measures how drastically the genes

of a certain pathway are overrepresented, which was calculated by the percentage of differently expressed proteins belonging to a pathway divided by the corresponding percentage in the background. FDR was calculated based on nominal P-value from the hypergeometric test, which represents the statistical probability of each enrichment. A cut-off of 0.05 was used on the FDR to filter enriched pathways. Calculations were performed using R\GO.db and R\Gostats packages, and visualizations were performed using R\ ggplot2.
