# Supplementary material for: Proteomics Analysis of Peripheral Blood Mononuclear Cells from Patients in Early Dengue Infection Reveals Potential Markers of Subsequent Fluid Leakage
Source: Viruses. 2025 May 31;17(6):805. doi: 10.3390/v17060805 (PMC12197526; doi:10.3390/v17060805)
Supplement: Supplementary file 1 [file viruses-17-00805-s001.zip › Supplementary Table S1.pdf]

Supplementary Table S1: Differentially expressed proteins in dengue-infected cell lysates compared to healthy controls.

Accession	Protein names	$\log_2$ fold change	$-\log_{10}$ p value
RAB8A	Ras-related protein Rab-8A (EC 3.6.5.2) (Oncogene c-mel)	-1.6408	4.51041
SYDC	Aspartate--tRNA ligase, cytoplasmic (EC 6.1.1.12) (Aspartyl-tRNA synthetase) (AspRS) (Cell proliferation-inducing gene 40 protein)	-1.1148	3.23091
CUTA	Protein CutA (Acetylcholinesterase-associated protein) (Brain acetylcholinesterase putative membrane anchor)	-1.0991	2.17585
TLN1	Talin-1	-1.0935	1.74936
RAB1B	Ras-related protein Rab-1B (EC 3.6.5.2)	-1.087	2.47392
FIBG	Fibrinogen gamma chain	-1.0096	2.48404
NOP56	Nucleolar protein 56 (Nucleolar protein 5A)	-1.0028	1.31267
DHB8	(3R)-3-hydroxyacyl-CoA dehydrogenase (EC 1.1.1.n12) (17-beta-hydroxysteroid dehydrogenase 8) (17-beta-HSD 8) (HSD17B8) (3-ketoacyl-[acyl-carrier-protein] reductase alpha subunit) (KAR alpha subunit) (3-oxoacyl-[acyl-carrier-protein] reductase) (Estradiol 17-beta-dehydrogenase 8) (EC 1.1.1.62) (Protein Ke6) (Ke6) (Short chain dehydrogenase/reductase family 30C member 1) (Testosterone 17-beta-dehydrogenase 8) (EC 1.1.1.239)	-1.0025	2.83755

PDIA5	Protein disulfide-isomerase A5 (EC 5.3.4.1) (Protein disulfide isomerase-related protein)	-0.9802	1.73983
RAC2	Ras-related C3 botulinum toxin substrate 2 (GX) (Small G protein) (p21-Rac2)	-0.9802	5.50963
PADI4	Protein-arginine deiminase type-4 (EC 3.5.3.15) (HL-60 PAD) (Peptidylarginine deiminase IV) (Protein-arginine deiminase type IV)	-0.9498	2.35834
ESYT1	Extended synaptotagmin-1 (E-Syt1) (Membrane-bound C2 domain-containing protein)	-0.9334	2.96682
G3P	Glyceraldehyde-3-phosphate dehydrogenase (GAPDH) (EC 1.2.1.12) (Peptidyl-cysteine S-nitrosylase GAPDH) (EC 2.6.99.-)	-0.9315	2.53363
IQGA2	Ras GTPase-activating-like protein IQGAP2	-0.931	4.82133
RB27A	Ras-related protein Rab-27A (Rab-27) (EC 3.6.5.2) (GTP-binding protein Ram)	-0.9278	4.36408
AP2B1	AP-2 complex subunit beta (AP105B) (Adaptor protein complex AP-2 subunit beta) (Adaptor-related protein complex 2 subunit beta) (Beta-2-adaptin) (Beta-adaptin) (Clathrin assembly protein complex 2 beta large chain) (Plasma membrane adaptor HA2/AP2 adaptin beta subunit)	-0.9235	1.35898
FA5	Coagulation factor V (Activated protein C cofactor) (Proaccelerin, labile factor) [Cleaved into: Coagulation factor V heavy chain; Coagulation factor V light chain]	-0.8998	3.95782

TRML1	Trem-like transcript 1 protein (TLT-1) (Triggering receptor expressed on myeloid cells-like protein 1)	-0.8985	3.3608
MYO1G	Unconventional myosin-Ig [Cleaved into: Minor histocompatibility antigen HA-2 (mHag HA-2)]	-0.8955	3.92792
F13A	Coagulation factor XIII A chain (Coagulation factor XIIIa) (EC 2.3.2.13) (Protein-glutamine gamma-glutamyltransferase A chain) (Transglutaminase A chain)	-0.8864	2.08252
ARC1B	Actin-related protein 2/3 complex subunit 1B (Arp2/3 complex 41 kDa subunit) (p41-ARC)	-0.8734	5.53607
PREX1	Phosphatidylinositol 3,4,5-trisphosphate-dependent Rac exchanger 1 protein (P-Rex1) (PtdIns(3,4,5)-dependent Rac exchanger 1)	-0.8689	3.54944
UBCP1	Ubiquitin-like domain-containing CTD phosphatase 1 (EC 3.1.3.16) (Nuclear proteasome inhibitor UBLCP1)	-0.8606	3.47106
KPYM	Pyruvate kinase PKM (EC 2.7.1.40) (Cytosolic thyroid hormone-binding protein) (CTHBP) (Opa-interacting protein 3) (OIP-3) (Pyruvate kinase 2/3) (Pyruvate kinase muscle isozyme) (Threonine-protein kinase PKM2) (EC 2.7.11.1) (Thyroid hormone-binding protein 1) (THBP1) (Tumor M2-PK) (Tyrosine-protein kinase PKM2) (EC 2.7.10.2) (p58)	-0.8575	2.96647

GNAQ	Guanine nucleotide-binding protein G(q) subunit alpha (Guanine nucleotide-binding protein alpha-q)	-0.857	1.89363
ICAL	Calpastatin (Calpain inhibitor) (Sperm BS-17 component)	-0.857	1.89363
STA5B	Signal transducer and activator of transcription 5B	-0.8402	4.41975
RDH11	Retinol dehydrogenase 11 (EC 1.1.1.300) (Androgen-regulated short-chain dehydrogenase/reductase 1) (HCV core-binding protein HCBP12) (Prostate short-chain dehydrogenase/reductase 1) (Retinal reductase 1) (RalR1) (Short chain dehydrogenase/reductase family 7C member 1)	-0.8383	4.4237
TCPG	T-complex protein 1 subunit gamma (TCP-1-gamma) (CCT-gamma) (hTRiC5)	-0.8379	2.33815
PPCS	Phosphopantothenate--cysteine ligase (EC 6.3.2.51) (Phosphopantothenoylcysteine synthetase) (PPC synthetase)	-0.8349	4.24995
HXK1	Hexokinase-1 (EC 2.7.1.1) (Brain form hexokinase) (Hexokinase type I) (HK I) (Hexokinase-A)	-0.8287	2.6769
KS6A3	Ribosomal protein S6 kinase alpha-3 (S6K-alpha-3) (EC 2.7.11.1) (90 kDa ribosomal protein S6 kinase 3) (p90-RSK 3) (p90RSK3) (Insulin-stimulated protein kinase 1) (ISPK-1) (MAP kinase-activated protein kinase 1b) (MAPK-activated protein kinase 1b) (MAPKAP kinase 1b) (MAPKAPK-1b) (Ribosomal S6 kinase 2) (RSK-2) (pp90RSK2)	-0.824	4.0348

PROP	Properdin (Complement factor P)	-0.7991	6.19936
IDHP	Isocitrate dehydrogenase [NADP], mitochondrial (IDH) (EC 1.1.1.42) (ICD-M) (IDP) (NADP(+)-specific ICDH) (Oxalosuccinate decarboxylase)	-0.7961	3.42847
PUR9	Bifunctional purine biosynthesis protein ATIC (AICAR transformylase/inosine monophosphate cyclohydrolase) (ATIC) [Cleaved into: Bifunctional purine biosynthesis protein ATIC, N-terminally processed] [Includes: Phosphoribosylaminoimidazolecarboxamide formyltransferase (EC 2.1.2.3) (5-aminoimidazole-4-carboxamide ribonucleotide formyltransferase) (AICAR formyltransferase) (AICAR transformylase); Inosine 5'-monophosphate cyclohydrolase (IMP cyclohydrolase) (EC 3.5.4.10) (IMP synthase) (Inosinicase)]	-0.7833	1.98293
TBA4A	Tubulin alpha-4A chain (EC 3.6.5.-) (Alpha-tubulin 1) (Testis-specific alpha-tubulin) (Tubulin H2-alpha) (Tubulin alpha-1 chain)	-0.7722	2.06768
KAPCA	cAMP-dependent protein kinase catalytic subunit alpha (PKA C-alpha) (EC 2.7.11.11)	-0.761	3.87938
UN13D	Protein unc-13 homolog D (Munc13-4)	-0.7564	4.25309
PARVB	Beta-parvin (Affixin)	-0.7544	2.50045
ANXA7	Annexin A7 (Annexin VII) (Annexin-7) (Synexin)	-0.7368	3.47118
PYGB	Glycogen phosphorylase, brain form (EC 2.4.1.1)	-0.7357	3.16318
DOCK2	Dedicator of cytokinesis protein 2	-0.7263	1.6351

AP2A1	AP-2 complex subunit alpha-1 (100 kDa coated vesicle protein A) (Adaptor protein complex AP-2 subunit alpha-1) (Adaptor-related protein complex 2 subunit alpha-1) (Alpha-adaptin A) (Alpha1-adaptin) (Clathrin assembly protein complex 2 alpha-A large chain) (Plasma membrane adaptor HA2/AP2 adaptin alpha A subunit)	-0.7255	2.0213
RS11	Small ribosomal subunit protein uS17 (40S ribosomal protein S11)	-0.7242	1.60808
DPM1	Dolichol-phosphate mannosyltransferase subunit 1 (EC 2.4.1.83) (Dolichol-phosphate mannose synthase subunit 1) (DPM synthase subunit 1) (Dolichyl-phosphate beta-D-mannosyltransferase subunit 1) (Mannose-P-dolichol synthase subunit 1) (MPD synthase subunit 1)	-0.7174	2.14828
AHNK	Neuroblast differentiation-associated protein AHNAK (Desmoyokin)	-0.7077	2.04483
RS4X	Small ribosomal subunit protein eS4, X isoform (40S ribosomal protein S4) (SCR10) (Single copy abundant mRNA protein)	-0.7069	1.78154
DYN2	Dynamin-2 (EC 3.6.5.5)	-0.7035	2.2243
H2AY	Core histone macro-H2A.1 (Histone macroH2A1) (mH2A1) (Histone H2A.y) (H2A/y) (Medulloblastoma antigen MU-MB-50.205)	-0.7024	1.57177
ITCH	E3 ubiquitin-protein ligase Itchy homolog (Itch) (EC 2.3.2.26) (Atrophin-1-interacting protein 4) (AIP4) (HECT-type E3 ubiquitin	-0.6923	3.72082

	transferase Itchy homolog) (NFE2-associated polypeptide 1) (NAPP1)		
VP13C	Intermembrane lipid transfer protein VPS13C (Vacuolar protein sorting-associated protein 13C)	-0.6922	4.62512
RHG01	Rho GTPase-activating protein 1 (CDC42 GTPase-activating protein) (GTPase-activating protein rhoGAP) (Rho-related small GTPase protein activator) (Rho-type GTPase-activating protein 1) (p50-RhoGAP)	-0.69	4.45492
SORL	Sortilin-related receptor (Low-density lipoprotein receptor relative with 11 ligand-binding repeats) (LDLR relative with 11 ligand-binding repeats) (LR11) (SorLA-1) (Sorting protein-related receptor containing LDLR class A repeats) (SorLA)	-0.6876	4.30552
MYLK	Myosin light chain kinase, smooth muscle (MLCK) (smMLCK) (EC 2.7.11.18) (Kinase-related protein) (KRP) (Telokin) [Cleaved into: Myosin light chain kinase, smooth muscle, deglutamylated form]	-0.6875	3.0512
RHG15	Rho GTPase-activating protein 15 (ArhGAP15) (Rho-type GTPase-activating protein 15)	-0.6801	2.09939
GNA13	Guanine nucleotide-binding protein subunit alpha-13 (G alpha-13) (G-protein subunit alpha-13)	-0.6794	2.65469
OSGEP	tRNA N6-adenosine threonylcarbamoyltransferase (EC 2.3.1.234) (N6-L-threonylcarbamoyladenine synthase) (t(6)A synthase) (O-	-0.6726	4.54695

	sialoglycoprotein endopeptidase) (hOSGEP) (t(6)A37 threonylcarbamoyladenosine biosynthesis protein OSGEP) (tRNA threonylcarbamoyladenosine biosynthesis protein OSGEP)		
CPNE1	Copine-1 (Chromobindin 17) (Copine I)	-0.6711	2.00788
LEG10	Galectin-10 (Gal-10) (Charcot-Leyden crystal protein) (CLC) (Eosinophil lysophospholipase) (Lysolecithin acylhydrolase)	-0.6633	2.70779
ABCD3	ATP-binding cassette sub-family D member 3 (EC 3.1.2.-) (EC 7.6.2.-) (70 kDa peroxisomal membrane protein) (PMP70)	-0.6588	1.48786
CO3	Complement C3 (C3 and PZP-like alpha-2-macroglobulin domain-containing protein 1) [Cleaved into: Complement C3 beta chain; C3-beta-c (C3bc); Complement C3 alpha chain; C3a anaphylatoxin; Acylation stimulating protein (ASP) (C3adesArg); Complement C3b alpha' chain; Complement C3c alpha' chain fragment 1; Complement C3dg fragment; Complement C3g fragment; Complement C3d fragment; Complement C3f fragment; Complement C3c alpha' chain fragment 2]	-0.6568	4.92904
PHB2	Prohibitin-2 (B-cell receptor-associated protein BAP37) (D-prohibitin) (Repressor of estrogen receptor activity)	-0.6551	2.8122
EIF3L	Eukaryotic translation initiation factor 3 subunit L (eIF3l) (Eukaryotic translation initiation factor 3 subunit 6-interacting	-0.6519	1.8001

	protein) (Eukaryotic translation initiation factor 3 subunit E-interacting protein)		
URP2	Fermitin family homolog 3 (Kindlin-3) (MIG2-like protein) (Unc-112-related protein 2)	-0.6504	3.07711
ERAP2	Endoplasmic reticulum aminopeptidase 2 (EC 3.4.11.-) (Leukocyte-derived arginine aminopeptidase) (L-RAP)	-0.6493	2.3333
MYO1F	Unconventional myosin-If (Myosin-Ie)	-0.647	3.14186
SPB10	Serpin B10 (Bomapin) (Peptidase inhibitor 10) (PI-10)	-0.6464	3.25896
HACD2	Very-long-chain (3R)-3-hydroxyacyl-CoA dehydratase 2 (EC 4.2.1.134) (3-hydroxyacyl-CoA dehydratase 2) (HACD2) (Protein-tyrosine phosphatase-like member B)	-0.6456	2.7587
MK01	Mitogen-activated protein kinase 1 (MAP kinase 1) (MAPK 1) (EC 2.7.11.24) (ERT1) (Extracellular signal-regulated kinase 2) (ERK-2) (MAP kinase isoform p42) (p42-MAPK) (Mitogen-activated protein kinase 2) (MAP kinase 2) (MAPK 2)	-0.6419	3.48089
K1H1	Keratin, type I cuticular Ha1 (Hair keratin, type I Ha1) (Keratin-31) (K31)	-0.6409	3.56327
LMNB2	Lamin-B2	-0.6345	2.26145
SYYC	Tyrosine--tRNA ligase, cytoplasmic (EC 6.1.1.1) (Tyrosyl-tRNA synthetase) (TyrRS) [Cleaved into: Tyrosine--tRNA ligase, cytoplasmic, N-terminally processed]	-0.6314	2.23647

FLOT2	Flotillin-2 (Epidermal surface antigen) (ESA) (Membrane component chromosome 17 surface marker 1)	-0.6303	3.41973
ACAP2	Arf-GAP with coiled-coil, ANK repeat and PH domain-containing protein 2 (Centaurin-beta-2) (Cnt-b2)	-0.6294	2.24623
SLFN5	Schlafen family member 5	-0.6241	2.18452
EHD3	EH domain-containing protein 3 (PAST homolog 3)	-0.6225	1.42641
ACTZ	Alpha-centractin (Centractin) (ARP1) (Actin-RPV) (Centrosome-associated actin homolog)	-0.6169	3.17786
TALDO	Transaldolase (EC 2.2.1.2)	-0.6138	3.1055
AL4A1	Delta-1-pyrroline-5-carboxylate dehydrogenase, mitochondrial (P5C dehydrogenase) (EC 1.2.1.88) (Aldehyde dehydrogenase family 4 member A1) (L-glutamate gamma-semialdehyde dehydrogenase)	-0.6107	1.70345
H2AV	Histone H2A.V (H2A.F/Z) (H2A.Z variant histone 2)	-0.6102	1.73564
ETFB	Electron transfer flavoprotein subunit beta (Beta-ETF)	-0.6037	1.96674
2AAA	Serine/threonine-protein phosphatase 2A 65 kDa regulatory subunit A alpha isoform (Medium tumor antigen-associated 61 kDa protein) (PP2A subunit A isoform PR65-alpha) (PP2A subunit A isoform R1-alpha)	-0.6023	4.04254
TCPZ	T-complex protein 1 subunit zeta (TCP-1-zeta) (Acute morphine dependence-related protein 2) (CCT-zeta-1) (HTR3) (Tcp20)	-0.5999	3.20628

AT2A3	Sarcoplasmic/endoplasmic reticulum calcium ATPase 3 (SERCA3) (SR Ca(2+)-ATPase 3) (EC 7.2.2.10) (Calcium pump 3)	-0.5996	2.65293
TSN14	Tetraspanin-14 (Tspan-14) (DC-TM4F2) (Transmembrane 4 superfamily member 14)	-0.5963	2.58299
GRAN	Grancalcin	-0.5952	2.48858
DOCK8	Dedicator of cytokinesis protein 8	-0.5946	1.53804
CAP1	Adenylyl cyclase-associated protein 1 (CAP 1)	-0.5932	3.26462
ACAD8	Isobutyryl-CoA dehydrogenase, mitochondrial (IBDH) (EC 1.3.8.5) (Activator-recruited cofactor 42 kDa component) (ARC42) (Acyl-CoA dehydrogenase family member 8) (ACAD-8)	-0.5925	5.55364
RS20	Small ribosomal subunit protein uS10 (40S ribosomal protein S20)	-0.5869	1.45783
ETHE1	Persulfide dioxygenase ETHE1, mitochondrial (EC 1.13.11.18) (Ethylmalonic encephalopathy protein 1) (Hepatoma subtracted clone one protein) (Sulfur dioxygenase ETHE1)	-0.5854	2.83856
VDAC2	Voltage-dependent anion-selective channel protein 2 (VDAC-2) (hVDAC2) (Outer mitochondrial membrane protein porin 2)	-0.5804	2.80619
STXB2	Syntaxin-binding protein 2 (Protein unc-18 homolog 2) (Unc18-2) (Protein unc-18 homolog B) (Unc-18B)	-0.5779	3.62576
CMPK2	UMP-CMP kinase 2, mitochondrial (EC 2.7.4.14) (Nucleoside-diphosphate kinase) (EC 2.7.4.6)	0.58505	2.30287

CYBP	Calcyclin-binding protein (CacyBP) (hCacyBP) (S100A6-binding protein) (Siah-interacting protein)	0.59727	2.10818
NCF1B	Putative neutrophil cytosol factor 1B (NCF-1B) (Putative SH3 and PX domain-containing protein 1B)	0.59912	2.59086
NCF1	Neutrophil cytosol factor 1 (NCF-1) (47 kDa autosomal chronic granulomatous disease protein) (47 kDa neutrophil oxidase factor) (NCF-47K) (Neutrophil NADPH oxidase factor 1) (Nox organizer 2) (Nox-organizing protein 2) (SH3 and PX domain-containing protein 1A) (p47-phox)	0.59912	2.59086
IGG1	Immunoglobulin gamma-1 heavy chain (Immunoglobulin gamma-1 heavy chain NIE)	0.6057	1.50324
DDB2	DNA damage-binding protein 2 (DDB p48 subunit) (DDBb) (Damage-specific DNA-binding protein 2) (UV-damaged DNA-binding protein 2) (UV-DDB 2)	0.61114	2.99062
PSB4	Proteasome subunit beta type-4 (26 kDa prosomal protein) (HsBPROS26) (PROS-26) (Macropain beta chain) (Multicatalytic endopeptidase complex beta chain) (Proteasome beta chain) (Proteasome chain 3) (HsN3)	0.61343	2.75272
ATP5L	ATP synthase subunit g, mitochondrial (ATPase subunit g) (ATP synthase membrane subunit g)	0.61531	1.56848
ATX10	Ataxin-10 (Brain protein E46 homolog) (Spinocerebellar ataxia type 10 protein)	0.63205	2.66142

PSB2	Proteasome subunit beta type-2 (Macropain subunit C7-I) (Multicatalytic endopeptidase complex subunit C7-I) (Proteasome component C7-I)	0.63278	2.23974
GSTO1	Glutathione S-transferase omega-1 (GSTO-1) (EC 2.5.1.18) (Glutathione S-transferase omega 1-1) (GSTO 1-1) (Glutathione-dependent dehydroascorbate reductase) (EC 1.8.5.1) (Monomethylarsonic acid reductase) (MMA(V) reductase) (EC 1.20.4.2) (S-(Phenacyl)glutathione reductase) (SPG-R)	0.64442	1.72378
SAM9L	Sterile alpha motif domain-containing protein 9-like (SAM domain-containing protein 9-like)	0.64443	1.98439
EST1	Liver carboxylesterase 1 (Acyl-coenzyme A:cholesterol acyltransferase) (ACAT) (Brain carboxylesterase hBr1) (Carboxylesterase 1) (CE-1) (hCE-1) (EC 3.1.1.1) (Cholesteryl ester hydrolase) (CEH) (EC 3.1.1.13) (Cocaine carboxylesterase) (Egasyn) (HMSE) (Methylumbelliferyl-acetate deacetylase 1) (EC 3.1.1.56) (Monocyte/macrophage serine esterase) (Retinyl ester hydrolase) (REH) (Serine esterase 1) (Triacylglycerol hydrolase) (TGH)	0.64642	3.02035
DDX58	DEAD box protein 58 (Antiviral innate immune response receptor RIG-1)	0.64919	2.49513
APEX1	DNA-(apurinic or apyrimidinic site) endonuclease (EC 3.1.11.2) (APEX nuclease) (APEN) (Apurinic-apyrimidinic endonuclease	0.6559	1.31424

	1) (AP endonuclease 1) (APE-1) (REF-1) (Redox factor-1) [Cleaved into: DNA-(apurinic or apyrimidinic site) endonuclease, mitochondrial]		
A1AT	Alpha-1-antitrypsin (Alpha-1 protease inhibitor) (Alpha-1-antiproteinase) (Serpin A1) [Cleaved into: Short peptide from AAT (SPAAT)]	0.65814	1.78611
S10A8	Protein S100-A8 (Calgranulin-A) (Calprotectin L1L subunit) (Cystic fibrosis antigen) (CFAG) (Leukocyte L1 complex light chain) (Migration inhibitory factor-related protein 8) (MRP-8) (p8) (S100 calcium-binding protein A8) (Urinary stone protein band A)	0.67491	1.72197
SAP	Prosaposin (Proactivator polypeptide) [Cleaved into: Saposin-A (Protein A); Saposin-B-Val; Saposin-B (Cerebroside sulfate activator) (CSAct) (Dispersin) (Sphingolipid activator protein 1) (SAP-1) (Sulfatide/GM1 activator); Saposin-C (A1 activator) (Co-beta-glucosidase) (Glucosylceramidase activator) (Sphingolipid activator protein 2) (SAP-2); Saposin-D (Component C) (Protein C)]	0.67557	1.3805
GCN1	Stalled ribosome sensor GCN1 (GCN1 eIF-2-alpha kinase activator homolog) (GCN1-like protein 1) (General control of amino-acid synthesis 1-like protein 1) (Translational activator GCN1) (HsGCN1)	0.68212	1.54991

HNRL2	Heterogeneous nuclear ribonucleoprotein U-like protein 2 (Scaffold-attachment factor A2) (SAF-A2)	0.69468	1.44467
DDX41	Probable ATP-dependent RNA helicase DDX41 (EC 3.6.4.13) (DEAD box protein 41) (DEAD box protein abstrakt homolog)	0.70937	2.3936
KDM1A	Lysine-specific histone demethylase 1A (EC 1.14.99.66) (BRAF35-HDAC complex protein BHC110) (Flavin-containing amine oxidase domain-containing protein 2) ([histone H3]-dimethyl-L-lysine(4) FAD-dependent demethylase 1A)	0.73111	1.61637
TIAR	Nucleolysin TIAR (TIA-1-related protein)	0.73732	2.06249
CALR	Calreticulin (CRP55) (Calregulin) (Endoplasmic reticulum resident protein 60) (ERp60) (HACBP) (grp60)	0.73835	3.33055
MYL6	Myosin light polypeptide 6 (17 kDa myosin light chain) (LC17) (Myosin light chain 3) (MLC-3) (Myosin light chain alkali 3) (Myosin light chain A3) (Smooth muscle and nonmuscle myosin light chain alkali 6)	0.74997	1.75992
CATD	Cathepsin D (EC 3.4.23.5) [Cleaved into: Cathepsin D light chain; Cathepsin D heavy chain]	0.75227	2.02338
TAP1	Antigen peptide transporter 1 (APT1) (EC 7.4.2.14) (ATP-binding cassette sub-family B member 2) (Peptide supply factor 1) (Peptide transporter PSF1) (PSF-1) (Peptide transporter TAP1) (Peptide transporter involved in antigen processing 1) (Really interesting new gene 4 protein) (RING4)	0.78453	2.82634

CALX	Calnexin (IP90) (Major histocompatibility complex class I antigen-binding protein p88) (p90)	0.79113	1.67873
PSB10	Proteasome subunit beta type-10 (EC 3.4.25.1) (Low molecular mass protein 10) (Macropain subunit MECl-1) (Multicatalytic endopeptidase complex subunit MECl-1) (Proteasome MECl-1) (Proteasome subunit beta-2i)	0.80007	2.8627
CATG	Cathepsin G (CG) (EC 3.4.21.20) [Cleaved into: Cathepsin G, C-terminal truncated form]	0.80272	3.05875
FILA	Filaggrin	0.8126	1.58315
PLS1	Phospholipid scramblase 1 (PL scramblase 1) (Ca(2+)-dependent phospholipid scramblase 1) (Erythrocyte phospholipid scramblase) (Mg(2+)-dependent nuclease) (EC 3.1.-.-) (MmTRA1b)	0.81489	3.94907
ARK72	Aflatoxin B1 aldehyde reductase member 2 (EC 1.1.1.n11) (AFB1 aldehyde reductase 1) (AFB1-AR 1) (Aldoketoreductase 7) (Succinic semialdehyde reductase) (SSA reductase)	0.82628	1.36502
TYPH	Thymidine phosphorylase (TP) (EC 2.4.2.4) (Gliostatin) (Platelet-derived endothelial cell growth factor) (PD-ECGF) (TdRPase)	0.84238	2.5585
THUM1	THUMP domain-containing protein 1	0.85235	1.68893
GRAH	Granzyme H (EC 3.4.21.-) (CCP-X) (Cathepsin G-like 2) (CTSGL2) (Cytotoxic T-lymphocyte proteinase) (Cytotoxic serine protease C) (CSP-C)	0.90969	2.01037

AMPL	Cytosol aminopeptidase (EC 3.4.11.1) (Cysteinylglycine-S-conjugate dipeptidase) (EC 3.4.13.23) (Leucine aminopeptidase 3) (LAP-3) (Leucyl aminopeptidase) (Peptidase S) (Proline aminopeptidase) (EC 3.4.11.5) (Prolyl aminopeptidase)	0.9149	4.56312
BST2	Bone marrow stromal antigen 2 (BST-2) (HM1.24 antigen) (Tetherin) (CD antigen CD317)	0.91809	2.00901
TRFE	Serotransferrin (Transferrin) (Beta-1 metal-binding globulin) (Siderophilin)	0.92589	1.30616
SYWC	Tryptophan--tRNA ligase, cytoplasmic (EC 6.1.1.2) (Interferon-induced protein 53) (IFP53) (Tryptophanyl-tRNA synthetase) (TrpRS) (hWRS) [Cleaved into: T1-TrpRS; T2-TrpRS]	0.95457	1.83803
STAT1	Signal transducer and activator of transcription 1-alpha/beta (Transcription factor ISGF-3 components p91/p84)	0.9692	2.63354
CD38	ADP-ribosyl cyclase/cyclic ADP-ribose hydrolase 1 (EC 3.2.2.-) (EC 3.2.2.6) (2'-phospho-ADP-ribosyl cyclase) (2'-phospho-ADP-ribosyl cyclase/2'-phospho-cyclic-ADP-ribose transferase) (EC 2.4.99.20) (2'-phospho-cyclic-ADP-ribose transferase) (ADP-ribosyl cyclase 1) (ADPRC 1) (Cyclic ADP-ribose hydrolase 1) (cADPR hydrolase 1) (T10) (CD antigen CD38)	0.9852	3.99935
OAS2	2'-5'-oligoadenylate synthase 2 ((2-5')oligo(A) synthase 2) (2-5A synthase 2) (EC	1.00021	4.11193

	2.7.7.84) (p69 OAS / p71 OAS) (p69OAS / p71OAS)		
AACT	Alpha-1-antichymotrypsin (ACT) (Cell growth-inhibiting gene 24/25 protein) (Serpin A3) [Cleaved into: Alpha-1-antichymotrypsin His-Pro-less]	1.02357	2.90383
LG3BP	Galectin-3-binding protein (Basement membrane autoantigen p105) (Lectin galactoside-binding soluble 3-binding protein) (Mac-2-binding protein) (MAC2BP) (Mac-2 BP) (Tumor-associated antigen 90K)	1.02765	3.55188
IFIT2	Interferon-induced protein with tetratricopeptide repeats 2 (IFIT-2) (ISG-54 K) (Interferon-induced 54 kDa protein) (IFI-54K) (P54)	1.04792	3.96266
1A03	HLA class I histocompatibility antigen, A alpha chain	1.06703	1.37253
ALBU	Albumin	1.09259	1.32339
TAP2	Antigen peptide transporter 2 (APT2) (EC 7.4.2.14) (ATP-binding cassette sub-family B member 3) (Peptide supply factor 2) (Peptide transporter PSF2) (PSF-2) (Peptide transporter TAP2) (Peptide transporter involved in antigen processing 2) (Really interesting new gene 11 protein) (RING11)	1.11687	3.3616
OAS1	2'-5'-oligoadenylate synthase 1 ((2-5')oligo(A) synthase 1) (2-5A synthase 1) (EC 2.7.7.84) (E18/E16) (p46/p42 OAS)	1.12718	2.7413
TLN2	Talin-2	1.15894	2.04804

FBX6	F-box only protein 6 (F-box protein that recognizes sugar chains 2) (F-box/G-domain protein 2)	1.15963	3.0199
PSB3	Proteasome subunit beta type-3 (Proteasome chain 13) (Proteasome component C10-II) (Proteasome theta chain)	1.27874	2.23182
OAS3	2'-5'-oligoadenylate synthase 3 ((2-5')oligo(A) synthase 3) (2-5A synthase 3) (EC 2.7.7.84) (p100 OAS) (p100OAS)	1.28759	4.33362
IFIT1	Interferon-induced protein with tetratricopeptide repeats 1 (IFIT-1) (Interferon-induced 56 kDa protein) (IFI-56K) (P56)	1.35593	4.16924
IFIT3	Interferon-induced protein with tetratricopeptide repeats 3 (IFIT-3) (CIG49) (ISG-60) (Interferon-induced 60 kDa protein) (IFI-60K) (Interferon-induced protein with tetratricopeptide repeats 4) (IFIT-4) (Retinoic acid-induced gene G protein) (P60) (RIG-G)	1.40951	4.86468
MX2	Interferon-induced GTP-binding protein Mx2 (Interferon-regulated resistance GTP-binding protein MxB) (Myxovirus resistance protein 2) (p78-related protein)	1.4203	4.72113
ISG15	Ubiquitin-like protein ISG15 (Interferon-induced 15 kDa protein) (Interferon-induced 17 kDa protein) (IP17) (Ubiquitin cross-reactive protein) (hUCRP)	1.5937	4.1169
PARP9	Protein mono-ADP-ribosyltransferase PARP9 (EC 2.4.2.-) (ADP-ribosyltransferase	1.65224	3.78095

	diphtheria toxin-like 9) (ARTD9) (B aggressive lymphoma protein) (Poly [ADP-ribose] polymerase 9) (PARP-9)		
IFM2	Interferon-induced transmembrane protein 2 (Dispanin subfamily A member 2c) (DSPA2c) (Interferon-inducible protein 1-8D)	1.66974	3.31843
IFM1	Interferon-induced transmembrane protein 1 (Dispanin subfamily A member 2a) (DSPA2a) (Interferon-induced protein 17) (Interferon-inducible protein 9-27) (Leu-13 antigen) (CD antigen CD225)	1.66974	3.31843
IFM3	Interferon-induced transmembrane protein 3 (Dispanin subfamily A member 2b) (DSPA2b) (Interferon-inducible protein 1-8U)	1.66974	3.31843
SN	Sialoadhesin (Sialic acid-binding Ig-like lectin 1) (Siglec-1) (CD antigen CD169)	1.67598	3.94234
TSP1	Thrombospondin-1 (Glycoprotein G)	1.77911	1.40843
MX1	Interferon-induced GTP-binding protein Mx1 (Interferon-induced protein p78) (IFI-78K) (Interferon-regulated resistance GTP-binding protein MxA) (Myxoma resistance protein 1) (Myxovirus resistance protein 1) [Cleaved into: Interferon-induced GTP-binding protein Mx1, N-terminally processed]	2.00146	3.06241
LBP	Lipopolysaccharide-binding protein (LBP)	2.05851	1.84323
