# Supplementary material for: Proteomics Analysis of Peripheral Blood Mononuclear Cells from Patients in Early Dengue Infection Reveals Potential Markers of Subsequent Fluid Leakage
Source: Viruses. 2025 May 31;17(6):805. doi: 10.3390/v17060805 (PMC12197526; doi:10.3390/v17060805)
Supplement: Supplementary file 1 [file viruses-17-00805-s001.zip › Supplementary Table S2.pdf]

Supplementary Table S2: Differentially expressed proteins in DHF cell lysates compared to DF.

Accession	Protein names	log_2 fold change	$-log_{10}$ p value
FIBG	Fibrinogen gamma chain	-1.3631	1.47598
NCKP1	Nck-associated protein 1 (NAP 1) (Membrane-associated protein HEM-2) (p125Nap1)	-1.255	1.53176
LMNB2	Lamin-B2	-1.1896	1.31612
MMRN1	Multimerin-1 (EMILIN-4) (Elastin microfibril interface located protein 4) (Elastin microfibril interfacer 4) (Endothelial cell multimerin) [Cleaved into: Platelet glycoprotein Ia*; 155 kDa platelet multimerin (p-155) (p155)]	-1.1584	3.05892
VASP	Vasodilator-stimulated phosphoprotein (VASP)	-1.1429	1.44952
ERAP2	Endoplasmic reticulum aminopeptidase 2 (EC 3.4.11.-) (Leukocyte-derived arginine aminopeptidase) (L-RAP)	-1.1225	1.83566
CAVN2	Caveolae-associated protein 2 (Cavin-2) (PS-p68) (Phosphatidylserine-binding protein) (Serum deprivation-response protein)	-1.0455	1.64039
URP2	Fermitin family homolog 3 (Kindlin-3) (MIG2-like protein) (Unc-112-related protein 2)	-1.0264	3.12948
SBDS	Ribosome maturation protein SBDS (Shwachman-Bodian-Diamond syndrome protein)	-1.0147	2.43506
VWF	von Willebrand factor (vWF) [Cleaved into: von Willebrand antigen 2 (von Willebrand antigen II)]	-0.9961	1.53487
ETFB	Electron transfer flavoprotein subunit beta (Beta-ETF)	-0.9822	1.4466
RAB8A	Ras-related protein Rab-8A (EC 3.6.5.2) (Oncogene c-mel)	-0.9776	1.70114

GTR14	Solute carrier family 2, facilitated glucose transporter member 14 (Glucose transporter type 14) (GLUT-14)	-0.9664	3.17961
GTR3	Solute carrier family 2, facilitated glucose transporter member 3 (Glucose transporter type 3, brain) (GLUT-3)	-0.9664	3.17961
ITB3	Integrin beta-3 (Platelet membrane glycoprotein IIIa) (GPIIIa) (CD antigen CD61)	-0.9395	1.33539
RAB1B	Ras-related protein Rab-1B (EC 3.6.5.2)	-0.9112	2.78043
COPD	Coatomer subunit delta (Archain) (Delta-coat protein) (Delta-COP)	-0.8954	1.55949
WASP	Actin nucleation-promoting factor WAS (Wiskott-Aldrich syndrome protein) (WASp)	-0.8855	2.51693
CTGF	Cellular communication network family member 2	-0.8791	1.43092
TAGL2	Transgelin-2 (Epididymis tissue protein Li 7e) (SM22-alpha homolog)	-0.8722	2.02525
PSMD3	26S proteasome non-ATPase regulatory subunit 3 (26S proteasome regulatory subunit RPN3) (26S proteasome regulatory subunit S3) (Proteasome subunit p58)	-0.8666	1.31039
MIC60	MICOS complex subunit MIC60 (Cell proliferation-inducing gene 4/52 protein) (Mitochondrial inner membrane protein) (Mitofilin) (p87/89)	-0.855	3.11809
TLN1	Talin-1	-0.8547	2.5701
FIBA	Fibrinogen alpha chain [Cleaved into: Fibrinopeptide A; Fibrinogen alpha chain]	-0.8432	1.67125
SNX2	Sorting nexin-2 (Transformation-related gene 9 protein) (TRG-9)	-0.8399	3.43217
DDX46	Probable ATP-dependent RNA helicase DDX46 (EC 3.6.4.13) (DEAD box protein 46) (PRP5 homolog)	-0.8093	2.13341

ILK	Integrin-linked protein kinase (EC 2.7.11.1) (59 kDa serine/threonine-protein kinase) (Beta-integrin-linked kinase) (ILK-1) (ILK-2) (p59ILK)	-0.8075	1.34749
NHRF1	Na(+)/H(+) exchange regulatory cofactor NHE-RF1 (NHERF-1) (Ezrin-radixin-moesin-binding phosphoprotein 50) (EBP50) (Regulatory cofactor of Na(+)/H(+) exchanger) (Sodium-hydrogen exchanger regulatory factor 1) (Solute carrier family 9 isoform A3 regulatory factor 1)	-0.8033	2.19932
SYYC	Tyrosine--tRNA ligase, cytoplasmic (EC 6.1.1.1) (Tyrosyl-tRNA synthetase) (TyrRS) [Cleaved into: Tyrosine--tRNA ligase, cytoplasmic, N-terminally processed]	-0.8001	1.64877
GNAQ	Guanine nucleotide-binding protein G(q) subunit alpha (Guanine nucleotide-binding protein alpha-q)	-0.7853	1.60054
OPA1	Dynamin-like 120 kDa protein, mitochondrial (EC 3.6.5.5) (Optic atrophy protein 1) [Cleaved into: Dynamin-like 120 kDa protein, form S1]	-0.7764	2.01221
PARVB	Beta-parvin (Affixin)	-0.7763	1.51957
GPV	Platelet glycoprotein V (GPV) (Glycoprotein 5) (CD antigen CD42d)	-0.7716	3.61414
SEPT6	Septin-6	-0.7716	1.58305
NSF1C	NSFL1 cofactor p47 (UBX domain-containing protein 2C) (p97 cofactor p47)	-0.7666	1.75665
GP1BA	Platelet glycoprotein Ib alpha chain (GP-Ib alpha) (GPIb-alpha) (GPIbA) (Glycoprotein Ibalpha) (Antigen CD42b-alpha) (CD antigen CD42b) [Cleaved into: Glycocalicin]	-0.7512	1.31745
ANXA7	Annexin A7 (Annexin VII) (Annexin-7) (Synexin)	-0.7509	3.05138
EZRI	Ezrin (Cytovillin) (Villin-2) (p81)	-0.7503	1.70886

RDH11	Retinol dehydrogenase 11 (EC 1.1.1.300) (Androgen-regulated short-chain dehydrogenase/reductase 1) (HCV core-binding protein HCBP12) (Prostate short-chain dehydrogenase/reductase 1) (Retinal reductase 1) (RalR1) (Short chain dehydrogenase/reductase family 7C member 1)	-0.7502	3.18262
RHOF	Rho-related GTP-binding protein RhoF (Rho family GTPase Rif) (Rho in filopodia)	-0.7496	1.55845
SF3A3	Splicing factor 3A subunit 3 (SF3a60) (Spliceosome-associated protein 61) (SAP 61)	-0.7466	2.1756
AT2A3	Sarcoplasmic/endoplasmic reticulum calcium ATPase 3 (SERCA3) (SR Ca(2+)-ATPase 3) (EC 7.2.2.10) (Calcium pump 3)	-0.7256	2.60096
ICAM2	Intercellular adhesion molecule 2 (ICAM-2) (CD antigen CD102)	-0.7204	1.78744
RALB	Ras-related protein Ral-B (EC 3.6.5.2)	-0.7122	2.17143
GCN1	Stalled ribosome sensor GCN1 (GCN1 eIF-2-alpha kinase activator homolog) (GCN1-like protein 1) (General control of amino-acid synthesis 1-like protein 1) (Translational activator GCN1) (HsGCN1)	-0.7098	1.35645
RB27B	Ras-related protein Rab-27B (EC 3.6.5.2) (C25KG)	-0.7075	1.44913
IF4A2	Eukaryotic initiation factor 4A-II (eIF-4A-II) (eIF4A-II) (EC 3.6.4.13) (ATP-dependent RNA helicase eIF4A-2)	-0.7053	1.3219
CIP4	Cdc42-interacting protein 4 (Protein Felic) (Salt tolerant protein) (hSTP) (Thyroid receptor-interacting protein 10) (TR-interacting protein 10) (TRIP-10)	-0.7047	1.63399
ITA2B	Integrin alpha-IIb (GPalpha IIb) (GPIIb) (Platelet membrane glycoprotein IIb) (CD antigen CD41) [Cleaved into: Integrin alpha-IIb heavy chain;	-0.7035	2.05273

	Integrin alpha-IIb light chain, form 1; Integrin alpha-IIb light chain, form 2]		
ECE1	Endothelin-converting enzyme 1 (ECE-1) (EC 3.4.24.71)	-0.6973	2.03304
EHD3	EH domain-containing protein 3 (PAST homolog 3)	-0.6893	1.68911
F13A	Coagulation factor XIII A chain (Coagulation factor XIIIa) (EC 2.3.2.13) (Protein-glutamine gamma-glutamyltransferase A chain) (Transglutaminase A chain)	-0.6787	2.85746
HSPB1	Heat shock protein beta-1 (HspB1) (28 kDa heat shock protein) (Estrogen-regulated 24 kDa protein) (Heat shock 27 kDa protein) (HSP 27) (Stress-responsive protein 27) (SRP27)	-0.6697	1.38016
RINI	Ribonuclease inhibitor (Placental ribonuclease inhibitor) (Placental RNase inhibitor) (Ribonuclease/angiogenin inhibitor 1) (RAI)	-0.6695	1.49174
COR1C	Coronin-1C (Coronin-3) (hCRNN4)	-0.6543	1.33242
AT2A2	Sarcoplasmic/endoplasmic reticulum calcium ATPase 2 (SERCA2) (SR Ca(2+)-ATPase 2) (EC 7.2.2.10) (Calcium pump 2) (Calcium-transporting ATPase sarcoplasmic reticulum type, slow twitch skeletal muscle isoform) (Endoplasmic reticulum class 1/2 Ca(2+) ATPase)	-0.6473	2.90478
ITB1	Integrin beta-1 (Fibronectin receptor subunit beta) (Glycoprotein IIa) (GPIIA) (VLA-4 subunit beta) (CD antigen CD29)	-0.6395	1.9509
ROA2	Heterogeneous nuclear ribonucleoproteins A2/B1 (hnRNP A2/B1)	-0.6334	1.61068
TPM4	Tropomyosin alpha-4 chain (TM30p1) (Tropomyosin-4)	-0.6312	1.32585

RL28	Large ribosomal subunit protein eL28 (60S ribosomal protein L28)	-0.6254	1.33041
BAP31	B-cell receptor-associated protein 31 (BCR-associated protein 31) (Bap31) (6C6-AG tumor-associated antigen) (Protein CDM) (p28)	-0.6241	2.68604
COPA	Coatomer subunit alpha (Alpha-coat protein) (Alpha-COP) (HEP-COP) (HEPCOP) [Cleaved into: Xenin (Xenopsin-related peptide); Proxenin]	-0.6241	2.44365
EM55	55 kDa erythrocyte membrane protein (p55) (Membrane protein, palmitoylated 1)	-0.6157	1.38376
HS90B	Heat shock protein HSP 90-beta (HSP 90) (Heat shock 84 kDa) (HSP 84) (HSP84)	-0.6156	1.32305
IRAK4	Interleukin-1 receptor-associated kinase 4 (IRAK-4) (EC 2.7.11.1) (Renal carcinoma antigen NY-REN-64)	-0.613	1.34218
PAK2	Serine/threonine-protein kinase PAK 2 (EC 2.7.11.1) (Gamma-PAK) (PAK65) (S6/H4 kinase) (p21-activated kinase 2) (PAK-2) (p58) [Cleaved into: PAK-2p27 (p27); PAK-2p34 (p34) (C-t-PAK2)]	-0.6001	2.00303
ATP5L	ATP synthase subunit g, mitochondrial (ATPase subunit g) (ATP synthase membrane subunit g)	-0.5998	1.76212
G6PD	Glucose-6-phosphate 1-dehydrogenase (G6PD) (EC 1.1.1.49)	-0.5983	2.29229
RS3A	Small ribosomal subunit protein eS1 (40S ribosomal protein S3a) (v-fos transformation effector protein) (Fte-1)	-0.597	1.46349
KAPCA	cAMP-dependent protein kinase catalytic subunit alpha (PKA C-alpha) (EC 2.7.11.11)	-0.5939	2.01732
NID1	Nidogen-1 (NID-1) (Entactin)	-0.5915	2.44218
CLH1	Clathrin heavy chain 1 (Clathrin heavy chain on chromosome 17) (CLH-17)	-0.5899	2.45898
DEK	Protein DEK	-0.5893	1.97392

ATP5I	ATP synthase subunit e, mitochondrial (ATPase subunit e) (ATP synthase membrane subunit e) [Cleaved into: ATP synthase subunit e, mitochondrial, N-terminally processed]	-0.5873	1.36113
RSU1	Ras suppressor protein 1 (RSP-1) (Rsu-1)	-0.5843	1.7062
TCPZ	T-complex protein 1 subunit zeta (TCP-1-zeta) (Acute morphine dependence-related protein 2) (CCT-zeta-1) (HTR3) (Tcp20)	-0.5811	1.34918
FLNA	Filamin-A (FLN-A) (Actin-binding protein 280) (ABP-280) (Alpha-filamin) (Endothelial actin-binding protein) (Filamin-1) (Non-muscle filamin)	-0.5795	2.14458
MK01	Mitogen-activated protein kinase 1 (MAP kinase 1) (MAPK 1) (EC 2.7.11.24) (ERT1) (Extracellular signal-regulated kinase 2) (ERK-2) (MAP kinase isoform p42) (p42-MAPK) (Mitogen-activated protein kinase 2) (MAP kinase 2) (MAPK 2)	-0.5775	2.01257
K1C9	Keratin, type I cytoskeletal 9 (Cytokeratin-9) (CK-9) (Keratin-9) (K9)	0.59726	1.99881
HORN	Hornerin	0.67201	2.98874
PSB2	Proteasome subunit beta type-2 (Macropain subunit C7-I) (Multicatalytic endopeptidase complex subunit C7-I) (Proteasome component C7-I)	0.70867	1.53764
K22E	Keratin, type II cytoskeletal 2 epidermal (Cytokeratin-2e) (CK-2e) (Epithelial keratin-2e) (Keratin-2 epidermis) (Keratin-2e) (K2e) (Type-II keratin Kb2)	0.74177	1.43604
NGAL	Neutrophil gelatinase-associated lipocalin (NGAL) (25 kDa alpha-2-microglobulin-related subunit of MMP-9) (Lipocalin-2) (Oncogene 24p3) (Siderocalin) (p25)	0.76008	2.44488

PSB3	Proteasome subunit beta type-3 (Proteasome chain 13) (Proteasome component C10-II) (Proteasome theta chain)	0.82007	1.31385
NCF1	Neutrophil cytosol factor 1 (NCF-1) (47 kDa autosomal chronic granulomatous disease protein) (47 kDa neutrophil oxidase factor) (NCF-47K) (Neutrophil NADPH oxidase factor 1) (Nox organizer 2) (Nox-organizing protein 2) (SH3 and PX domain-containing protein 1A) (p47-phox)	0.82749	2.50284
NCF1B	Putative neutrophil cytosol factor 1B (NCF-1B) (Putative SH3 and PX domain-containing protein 1B)	0.82749	2.50284
ARI2	E3 ubiquitin-protein ligase ARIH2 (ARI-2) (Protein ariadne-2 homolog) (EC 2.3.2.31) (RING-type E3 ubiquitin transferase ARIH2) (Triad1 protein)	0.82933	1.72245
S10A9	Protein S100-A9 (Calgranulin-B) (Calprotectin L1H subunit) (Leukocyte L1 complex heavy chain) (Migration inhibitory factor-related protein 14) (MRP-14) (p14) (S100 calcium-binding protein A9)	0.85903	1.93371
S10A8	Protein S100-A8 (Calgranulin-A) (Calprotectin L1L subunit) (Cystic fibrosis antigen) (CFAG) (Leukocyte L1 complex light chain) (Migration inhibitory factor-related protein 8) (MRP-8) (p8) (S100 calcium-binding protein A8) (Urinary stone protein band A)	0.93615	1.76402
BPI	Bactericidal permeability-increasing protein (BPI) (CAP 57)	0.98905	1.69293
