# Supplementary figures and images for: Proteomics Analysis of Peripheral Blood Mononuclear Cells from Patients in Early Dengue Infection Reveals Potential Markers of Subsequent Fluid Leakage
Source: Viruses. 2025 May 31;17(6):805. doi: 10.3390/v17060805 (PMC12197526; doi:10.3390/v17060805)

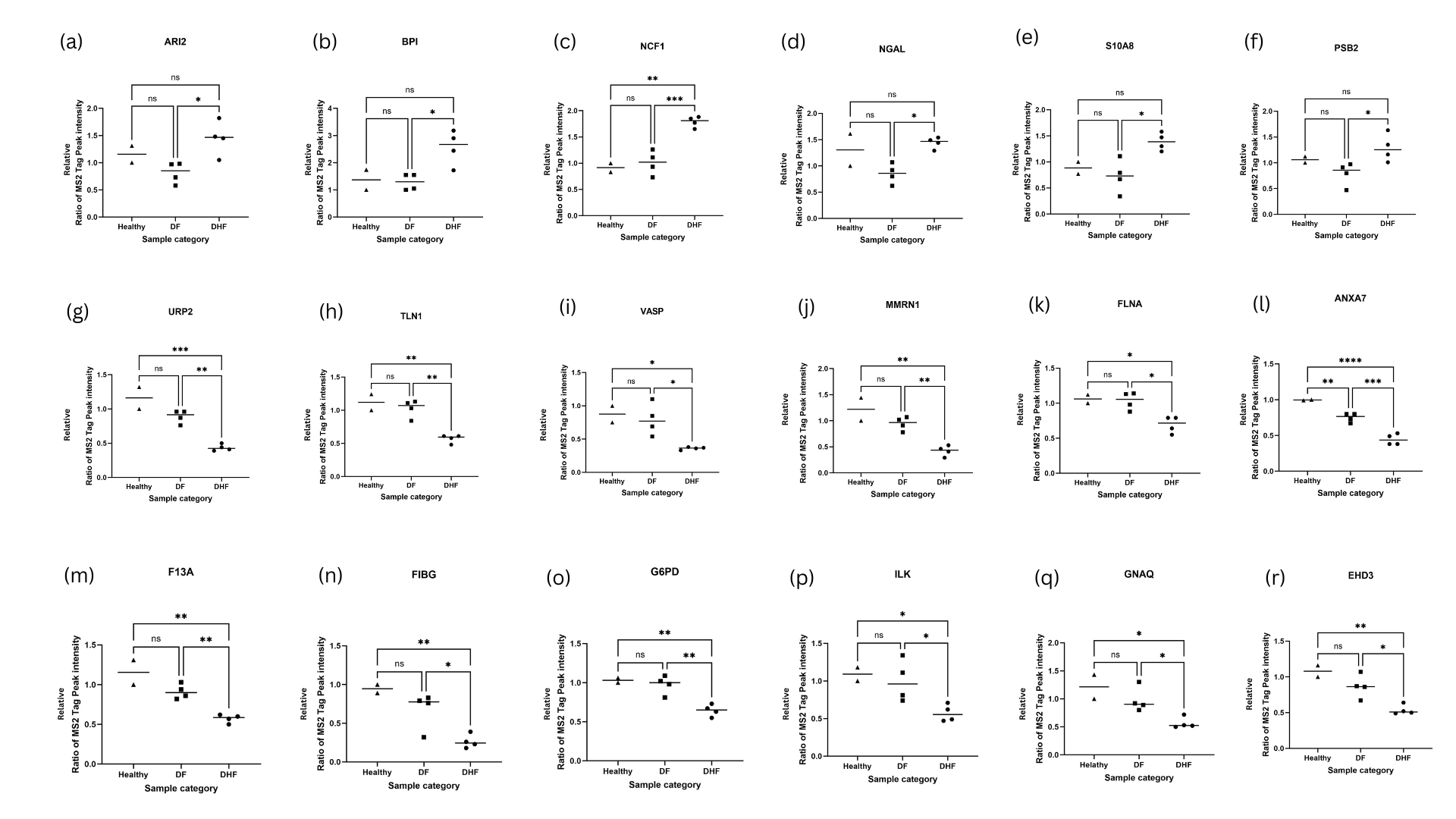

Supplement: Supplementary file 1 [file viruses-17-00805-s001.zip › Supplementary Figure S1.png]
